# Inflammation and Immune Mediators in the Context of Atherosclerotic Cardiovascular Disease and Percutaneous Coronary Intervention: Implications for Therapeutics

**DOI:** 10.3390/medsci14040430

**Published:** 2026-07-25

**Authors:** Karthik Chandrasekharan, Pratyasha Saha, Ioannis Merinopoulos, Simon Eccleshall, James G. W. Smith, Vassilios S. Vassiliou

**Affiliations:** 1 Department of Cardiology, Norfolk and Norwich University Hospital, Norwich NR4 7UY, UK; k.chandrasekharan@uea.ac.uk (K.C.);; 2 Norwich Medical School, University of East Anglia, Norwich NR4 7TJ, UK

**Keywords:** inflammation, atherosclerosis, colchicine, percutaneous coronary intervention

## Abstract

Atherosclerotic cardiovascular disease (CVD) remains a leading cause of worldwide morbidity and mortality, despite well-established/effective pharmacological therapies. A substantial component of the residual risk is inflammatory. The importance of inflammation and immune mediators in the pathophysiology has been recognised over the past two decades and, importantly, treatment with percutaneous coronary intervention (PCI) also results in an inflammatory response and associated adverse outcomes. Multiple biochemical pathways have been implicated with various identified biomarkers, such as high-sensitivity C-reactive protein which is now recognised as a marker of cardiovascular risk. Proteins such as interleukins (IL) IL-1 and IL-6 or larger protein complexes such as the NLRP3 inflammasome all play a key role in both the pathophysiology of atherosclerosis and inflammation relating to PCI. This knowledge has led to advances in pharmacological therapies and multiple pre-clinical and clinical trials, but to date, colchicine is the only treatment with sufficiently robust evidence from large-scale clinical trials to be recommended in multinational guidelines. Other therapies at present lack this level of evidence, and identification of other potential therapies is an important next step in treating inflammation post-PCI. Furthermore, the potential routine use of colchicine periprocedurally for PCI and its biochemical and clinical effects in this context are not yet fully established, requiring further investigation to identify its effect on specific inflammatory mediators and whether this translates to clinical outcomes.

## 1. Introduction

Cardiovascular disease (CVD) is of major significance since it is the leading cause of death in males in the UK and the second leading cause of death in the UK overall [1]. There is also significant economic impact, with a recent model estimating a total cost in the UK of over GDP 29 billion [2]. Atherosclerosis, via the development of plaques consisting primarily of cholesterol in arterial subendothelium, plays a key role in the development of CVD [3]. While traditional risk factors for the development of atherosclerosis, such as total cholesterol, hypertension, diabetes, and smoking, are well known, the importance of more novel risk factors such as inflammation (measured via markers such as high-sensitivity C-reactive protein (hs-CRP)) has become increasingly recognised [4]. Despite well-established lipid-lowering, antihypertensive, and anti-hyperglycaemic therapies, CVD is still the leading cause of death globally, necessitating additional treatment strategies such as those targeting inflammation [5]. Percutaneous coronary intervention (PCI) is important in the treatment of coronary artery disease (CAD), and importantly, PCI itself elicits an inflammatory response via a number of pathways, raising the possibility that anti-inflammatory medications such as colchicine may be beneficial periprocedurally [6]. Therefore, chronic inflammation as a risk factor for CAD and acute inflammation as a result of PCI should be considered separately with respect to therapeutic options. This narrative review will therefore explore the following. (1) The role of residual inflammatory risk in the pathogenesis of CAD. (2) The mechanisms and importance of acute periprocedural inflammation in the context of PCI. (3) Current evidence for anti-inflammatory therapies in the treatment of CAD and the potential therapeutic implications of modulating inflammation in patients undergoing PCI.

## 2. Materials and Methods

This article was conducted as a narrative review. PubMed was non-systematically searched to identify key publications relating to inflammation in atherosclerotic cardiovascular disease and the inflammatory response associated with percutaneous coronary intervention. Search terms included combinations of terms relating to atherosclerosis, coronary artery disease, percutaneous coronary intervention, inflammation, inflammatory biomarkers, immune pathways, and anti-inflammatory therapies.

The literature considered included original research articles, narrative reviews, systematic reviews, and meta-analyses. Relevant clinical guidelines and major clinical trials were also considered where applicable. Publications were selected according to their relevance to the aims of the review, their contribution to understanding the underlying mechanisms, and their clinical or therapeutic importance. The final manuscript was based on the articles considered by the authors to provide the most useful and important evidence for the topics discussed.

## 3. Inflammation and Pathogenesis of Atherosclerotic Cardiovascular Disease

The growing relevance of inflammation in the context of atherosclerosis and the subsequent development of CAD is widely acknowledged, with increasing appreciation in the last two decades [7,8,9]. Inflammation is known to play a significant role in the pathogenesis of atherosclerosis, involving a number of immune mediators, such as the balance between pro-inflammatory and anti-inflammatory macrophages affecting plaque progression or regression [10]. Links between specific biomarkers of inflammation such as hs-CRP or interleukin (IL)-6 and CAD have been identified, with recent evidence that higher hs-CRP levels were a stronger predictor than low-density lipoprotein cholesterol (LDL-C) of major adverse cardiovascular events (MACEs) and cardiovascular death in patients taking statins [11,12,13,14].

The innate immune system is involved in pathogenesis via both humoral and cellular pathways. Chemokines such as CCL2 secreted by activated endothelial cells attract monocytes to arteries. Monocytes eventually differentiate to form foam cells, as well as phagocytosing cholesterol crystals and oxidised lipids, which results in the activation of the inflammasome, specifically the nucleotide-binding oligomerisation domain-like receptors pyrin domain-containing 3 (NLRP3) inflammasome, leading to the activation of caspase-1 and the subsequent secretion of IL-1 [15,16,17].

These specific downstream inflammatory cytokines are vital in the pathogenesis of CAD, such as IL-1β, which has an important role in the development of atherosclerosis, and IL-18, which is involved in plaque destabilisation [18,19]. Other downstream cytokines such as IL-6 also demonstrate a correlation with the extent of CAD in acute coronary syndrome (ACS) patients [20]. Leukocytes have also been shown to be linked to atherosclerotic cardiovascular disease. Increased neutrophil count is associated with higher risk of cardiovascular death as well as non-fatal CVD [21]. Furthermore, monocytes have a role in atherosclerosis initiation, plaque destabilisation, and cardiac remodelling, with all monocyte subsets involved in pathogenesis [22].

Monocytes are the key precursor cells driving inflammatory processes in atherosclerosis [23]. There is now standardised nomenclature to classify monocyte subsets, depending on cell surface antigen expression: classical CD14++CD16−, monocytes, intermediate CD14++CD16+ monocytes, and non-classical CD14+CD16++ monocytes [24]. Classical monocytes (expressing CCR2, CD62L, and CD64) are the most prevalent circulating type in humans and are the predominant subtype found in atherosclerotic lesions, but non-classical monocytes (expressing high levels of CX3CR1) cause atherogenesis in murine models [22].

The relative expression of monocyte subsets appears to be significant, though there have been contrasting findings relating to classical monocytes, with some evidence that increased levels are associated with an increased risk of ischaemic cardiovascular events but not the baseline extent of atherosclerosis, whereas another study has shown no association between classical monocyte levels and vulnerable carotid plaque [25,26]. Non-classical monocytes also are important, with evidence of significant expansion in patients with CAD [27]. Finally, intermediate monocytes appear to play a significant role, with a prospective study of 951 individuals referred for elective angiography demonstrating that while both classical and intermediate monocytes predicted the primary endpoint of cardiovascular death, acute MI, or non-haemorrhagic stroke, only intermediate monocytes were independent predictors after adjusting for confounders [28]. Additionally, patients with CAD not only showed higher levels of intermediate monocytes, but also a shift in non-classical and classical monocytes towards intermediate monocytes, with additional evidence that intermediate monocytes are independently associated with MACE in individuals with established CAD [29,30]. Furthermore, individuals with vulnerable plaques identified on computed tomography show an increased relative proportion of CD14+CD16+ monocytes [31].

The adaptive immune system via both humoral and cellular pathways is also involved in atherogenesis. CD4+ T helper cells promote atherogenesis, with a mouse knockout model demonstrating a significant reduction in fatty streak lesions in immunodeficient mice, with a subsequent transfer of CD4+ T cells resulting in a marked increase in lesions [32]. Two particular subsets of T helper cells are particularly important: firstly, TH1 cells (which mediate immune responses via IFNγ) are the most abundant T helper cells found within atherosclerotic lesions, and secondly, TH17 cells (which mediate immune responses via IL-17A) are elevated in patients with ACS [33]. Additionally, activated regulatory T cells appear to be protective, with promotion of plaque stability and regression mediated via immunosuppressive cytokine TGF-β [34]. B cells are also involved in both development of CAD and protection against CAD, depending on the subset. IgM-producing B1 cells confer an anti-inflammatory and protective effect on atherosclerosis; contrastingly IgG-producing B2 cells result in an inflammatory response and are associated with CAD; and finally, Il-10-producing regulatory B cells (Bregs) are proposed to suppress CAD [35].

## 4. Inflammation and PCI

**Figure 1:**
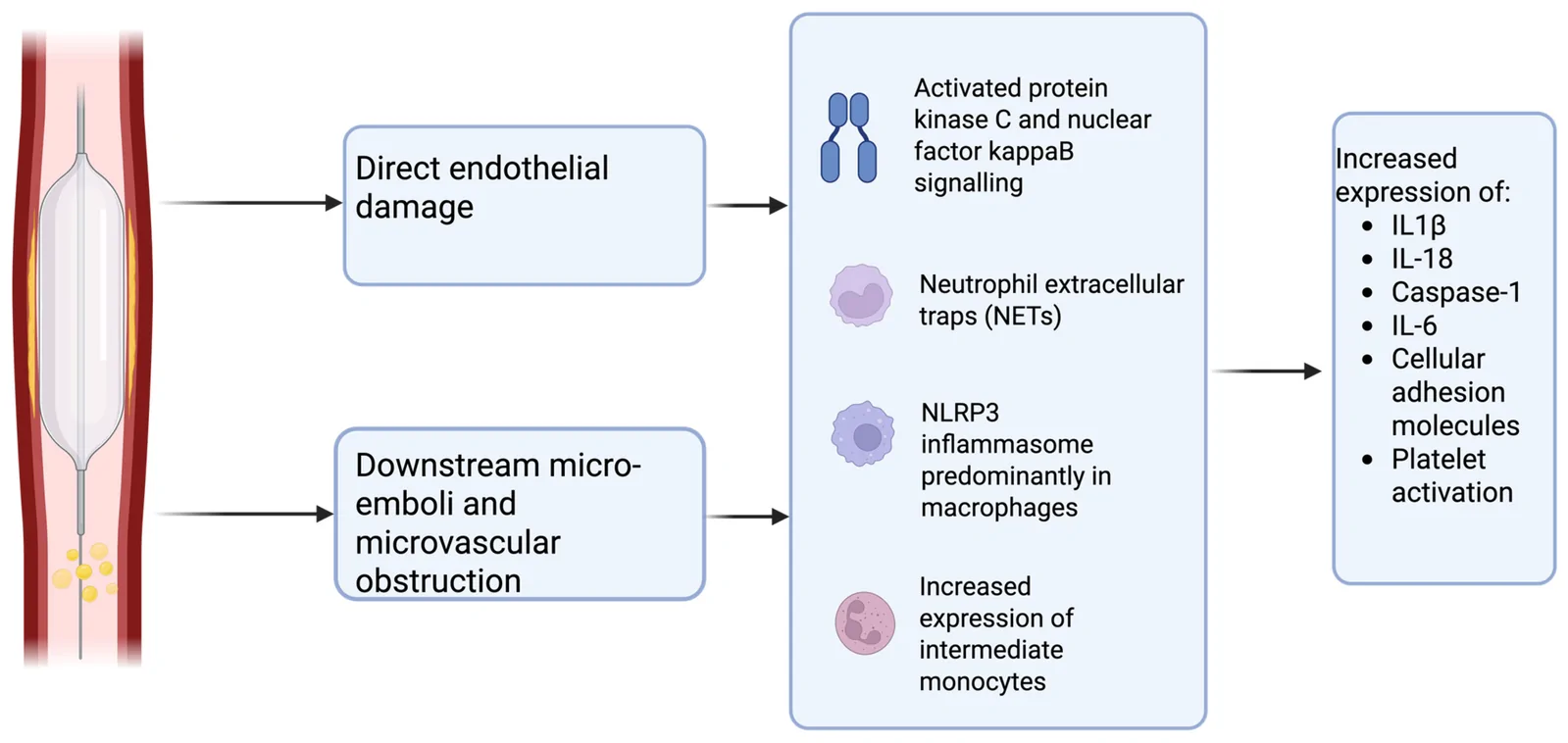
Overview of mechanisms of inflammation during PCI. Created in BioRender. Chandrasekharan, K. (2026) https://BioRender.com/y771h7h accessed on 12 July 2026.

Inflammation during PCI is a result of direct mechanical trauma induced by balloon inflation or stent deployment, both directly affecting the coronary endothelium and also disrupting coronary artery plaques, leading to microembolisation and release of pro-inflammatory material [6]. This is summarised in Figure 1. This has been demonstrated by significant increases in markers of endothelial damage such as von Willebrand factor, E-selectin, and circulating endothelial cells, identified within 15 min after elective PCI [36].

Inflammation in the periprocedural PCI setting is known to affect clinical outcomes, with a large-scale meta-analysis demonstrating that there is a 12% increase in risk of MACE for each 1 mg/L increment in pre-procedural serum CRP [37]. Inflammation is also associated with the pathogenesis of a number of adverse events following percutaneous coronary intervention (PCI), and markers of periprocedural inflammation such as higher levels of hs-CRP or elevated leukocyte count confer a higher risk of myocardial infarction, coronary revascularisation, restenosis, or early stent thrombosis [37,38,39,40,41]. The effects of periprocedural inflammation have been summarised by Tucker et al., with multiple studies demonstrating an association between elevated periprocedural hs-CRP and leukocyte count, with a number of adverse outcomes, particularly periprocedural MI and coronary no-reflow [6]. Acute inflammation, particularly via activation of the NLRP3 inflammasome following PCI for acute MI, also leads to the progression and vulnerability of non-culprit vessel CAD [42]. Inflammatory cytokines, platelet activation, and neutrophil infiltration are also involved in the mechanism of the no-reflow phenomenon, which is also a significant contributor to adverse outcomes following PCI [43]. A meta-analysis of 2747 patients undergoing PCI with a bare metal stent (BMS) showed that a higher pre-procedural CRP level was associated with significantly increased risk of in-stent restenosis [44].

There is contrasting evidence regarding the complexity of PCI or specific procedural techniques and the degree of inflammatory response, with one study of 75 individuals undergoing PCI with BMS or DES concluding that PCI of higher-risk coronary lesions, such as in-stent restenosis, coronary bifurcations, or total occlusions, results in a greater inflammatory response [45]. However, a more recent study has found that there is an acute reduction in pro-inflammatory cytokines, such as IL-1β, and an increase in anti-inflammatory IL-10 following PCI for chronic total occlusion [46]. Additionally, an increased number of stents does not lead to an increased level of inflammatory biomarkers such as IL-6, IL-1β, or CRP [47].

While PCI in general via the mechanisms outlined the results in an inflammatory response, it is important to note that ACS itself leads to a significant inflammatory response; while this is partly protective, for instance via Treg pathways, there is an acute localised inflammatory response primarily mediated via M1 macrophages and polymorphonuclear neutrophils resulting in increased infarct size and adverse remodelling, followed by a more residual low-grade inflammation via foam cells and the NLRP3 inflammasome as mentioned, which leads to increased risk of recurrent events and further atherogenesis [48]. Therefore, individuals undergoing PCI in the context of ACS may be at risk of a greater inflammatory response due to increased inflammasome activation [6].

Similar to the pathogenesis of atherosclerosis, the innate and adaptive immune system via both humoral and cellular pathway mediators are activated by PCI. Another acute phase reactant in the same family as CRP is pentraxin-3, which has recently emerged as a biomarker for cardiovascular inflammation, and elevated levels of pentraxin-3 post-PCI in patients with stable angina are associated with an increased risk of MACE during a 9-month follow up [49,50]. Downstream cytokines such as IL-6 have also been detected rapidly post-PCI [51,52,53]. An increase in IL-6 both acutely and after 4 months in patients undergoing PCI in the context of STEMI is associated with all-cause mortality, larger infarct size, and reduced LVEF [54].

Haematological parameters are also important markers of inflammatory risk. Although non-specific, elevated leukocyte levels are an independent predictor of periprocedural MI [55].

Circulating monocytes following PCI also appear to be significant. This was identified over two decades ago, with not only an increased monocyte count after the implantation of bare metal stents, but also an independent correlation with maximum monocyte count and in-stent neointimal volume [56]. Additionally, a lower lymphocyte:monocyte ratio is associated with a higher risk of ‘no-reflow’ phenomenon during PCI for either NSTEMI or STEMI [57,58].

The specific intervention also affects the inflammatory response, with platelet and neutrophil activation being greater following PCI for stable angina with bare metal stents compared with balloon angioplasty [59]. More recently, it has been shown that not only are intermediate monocyte levels significantly elevated two months following elective PCI, but they remain significantly elevated in patients treated with DES when compared with drug-coated balloon (DCB) angioplasty [60].

The NLRP3 inflammasome is an important regulator of periprocedural inflammation, with an increase in expression of genes composing this inflammasome, as well as an increase in IL-1B, IL-18, and caspase-1 correlating with periprocedural myocardial injury in patients undergoing elective PCI [61]. Gene expression of IL-1B and IL-18 is also elevated in the first 24 h in individuals presenting with STEMI who have undergone PCI [62].

Neutrophils are an important mediator of the periprocedural inflammatory response, releasing pro-inflammatory materials post-PCI including neutrophil extracellular traps (NETs), resulting in inflammatory cytokine release from T-lymphocytes, macrophages, and activated endothelial cells [6,63].

T cell response is also modulated by PCI, with TH1 and TH17 cell levels both increased immediately following PCI for ACS, but they decline after 1 month, whereas Tregs remain stable immediately following PCI but are elevated after 1 month [64].

## 5. Implications for Pharmacological Therapy

**Figure 2:**
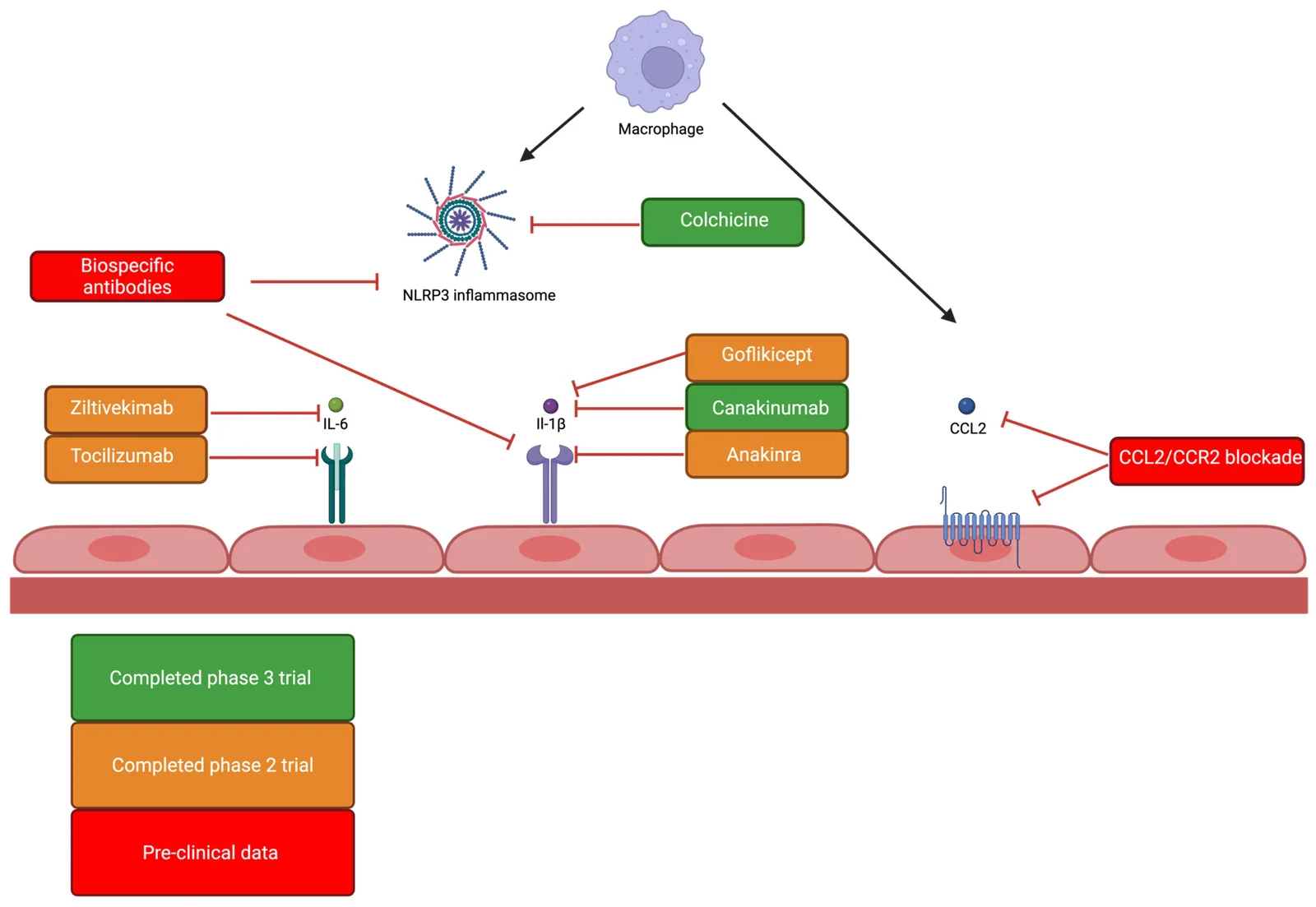
Summary of anti-inflammatory drug targets in the context of CAD and the phase of the trial completed. Created in BioRender. Chandrasekharan, K. (2026) https://BioRender.com/y771h7h accessed on 12 July 2026.

Translating the understanding of inflammation in the development of atherosclerotic disease to targeted pharmacological therapies can be challenging. Difficulty arises as there are numerous inflammatory pathways involved, some of which may also regulate anti-atherogenic processes, and therefore, targeting one specific pathway may not effectively disrupt the inflammatory cascade [65]. Well-established pharmacological treatments for CAD such as high-dose HMG-CoA reductase inhibitors (statins), proprotein convertase subtilisin/kexin type 9 (PCSK9) monoclonal antibodies (e.g., evolocumab and alirocumab), aspirin, and angiotensin-converting enzyme inhibitors (ACEi) or angiotensin-1 receptor blockers (ARBs) have all been shown to confer potential anti-inflammatory effects via numerous mechanisms [65]. Elevated hs-CRP may also identify individuals who will respond better to PCSK9 monoclonal antibody therapy, though interestingly, siRNA targeting PCSK9 (inclisiran), but not PCSK9 monoclonal antibodies themselves, reduces hs-CRP levels [66]. However, more recent progress has been made in targeted anti-inflammatory therapies, following large-scale randomised controlled trial results, as outlined in Figure 2.

Colchicine

Colchicine is an established treatment for numerous inflammatory conditions such as gout, pericarditis, and familial Mediterranean fever [67]. The mechanism of action of colchicine primarily involves binding to tubulins and inhibiting the assembly and polymerisation of microtubules, but other identified mechanisms of action include the inhibition of neutrophil chemotaxis and a reduction in IL-6 and hs-CRP [68]. However, there appears to be a significant novel mechanism of action that involves blocking the activity of the NLRP3 inflammasome, though the specific pathway remains uncertain, with the inhibition of monocyte cytokine production and the suppression of IL-1β and caspase-1 activity [69,70]. In patients with a history of MI, colchicine seems to exert its effect primarily by affecting neutrophil function, with a significant decrease in neutrophil recruitment, and its effect on monocytes is limited to a decrease in the HLA-DR cell surface receptor on intermediate and non-classical monocytes; there is no effect on the RNA expression of monocyte phenotypes [71]. Additionally, in non-cardiovascular disease settings, colchicine affects leukocyte populations including a reduction in classical monocytes and an increase in intermediate subsets [72,73].

In recent years, numerous large-scale clinical trials have suggested that colchicine confers clinical benefit in CAD, in a range of clinical scenarios including ACS, chronic coronary syndromes, and the periprocedural PCI setting. The COLCOT trial was a randomised double-blind trial, which randomised 4745 patients within 30 days of a myocardial infarction to receive either low-dose colchicine (0.5 mg once daily) or a placebo, and concluded that over a median follow up of 22.6 months, there was a significant reduction in the primary outcome of a composite of death for cardiovascular causes, resuscitated cardiac arrest, myocardial infarction (MI), stroke, or urgent hospitalisation for angina resulting in coronary revascularisation [74]. A secondary analysis has shown the introduction of colchicine within 3 days after myocardial infarction conferred a higher reduction in MACE at a median follow up of 22.7 months [75]. However, the more recent CLEAR trial found no reduction in cardiovascular events following colchicine administration, having randomised 7062 patients with a recent MI to receive either colchicine or placebo, and either spironolactone or placebo; there was no reduction in the incidence of the composite primary outcome of death for cardiovascular cause, recurrent MI, stroke, or unplanned ischaemia-driven coronary revascularisation after a median follow up of 3 years [76]. In contrast to these studies investigating the use of colchicine in the context of recent ACS, the LoDoCo2 trial was a randomised double-blind trial, which randomised 5522 patients with chronic coronary disease that had been stable for at least 6 months, to receive either low-dose colchicine (0.5 mg once daily) or a placebo, concluded that over a median follow up of 28.6 months, there was a significant reduction in the composite primary outcome of cardiovascular death spontaneous MI, ischaemic stroke, or ischaemia-driven coronary revascularisation [77]. Meta-analyses have also concluded that while low-dose colchicine reduces the risk of major adverse cardiovascular events and the need for coronary revascularisation, there was no significant difference in all-cause mortality [78].

Following these results, the use of colchicine for CAD is now supported in guidelines such as the 2023 European Society of Cardiology (ESC) guidelines for ACS, suggesting that low-dose colchicine (0.5 mg once daily) may be considered, especially if other risk factors are suboptimally controlled or cardiovascular events occur despite optimal therapy (Class IIb recommendation) [79]. The 2024 ESC guidelines for the management of chronic coronary syndromes also support the consideration of low-dose colchicine (0.5 mg once daily) in patients with atherosclerotic CAD to reduce myocardial infarction, stroke, and the need for revascularisation (Class IIa recommendation) [80].

Colchicine does have known side effects such as early gastrointestinal intolerance in around 10% of individuals receiving 0.5 mg daily, but if individuals are tolerant during therapy initiation, there is good long-term tolerance with infrequent late discontinuation, with low-dose colchicine achieving its clinical benefits at a serum level below the upper limit of safety in patients without advance renal or liver disease, and with no increase in the risk of cancer, sepsis, cytopaenia, or myotoxicity [81]. Additionally, a recent systematic review and meta-analysis has shown that colchicine was well tolerated with no increased risk of discontinuation in cardiovascular trials [82].

Colchicine is not routinely recommended periprocedurally for PCI, though this is an area of significant interest across several randomised trials. The use of colchicine periprocedurally in the context of PCI has been shown to reduce NET release in ACS patients undergoing PCI within 24 h of treatment [63]. The COPE-PCI trial was a small pilot trial that randomised 75 patients to receive a total of 1.5 mg colchicine or a placebo 6–24 h prior to PCI, and it found that there was a significant reduction in periprocedural myocardial injury as measured by hs-troponin levels [83]. Contrastingly, however, the COLCHICINE-PCI trial randomised 400 patients who underwent PCI to receive either 1.8 mg of colchicine or placebo prior to PCI within one to two hours prior to angiography, and it found that while there was no reduction in the risk of periprocedural myocardial injury, there was a significant attenuation in the rise in IL-6 and hs-CRP 24 h post-PCI [84]. The utility of colchicine in the context of STEMI has also been investigated in the PodCAST-PCI trial, which randomised 321 patients admitted with acute STEMI to receive 1 mg colchicine prior to PCI and 0.5 mg daily until discharge, or a placebo, finding no difference in P-selectin, hs-CRP, or troponin between the two groups [85].

Therefore, it is clear that there are conflicting results across multiple trials investigating the use of colchicine as summarised in Table 1, both with regard to its use in the context of ACS, given the contrasting results of COLCOT and CLEAR trials, as well as in the context of modulating periprocedural inflammation post-PCI. It is important to note the difference in the timing of colchicine administration relating to PCI between trials, and whether this may account for the conflicting results with respect to periprocedural myocardial injury, as well as a potential difference due to the baseline inflammatory burden of ACS. Further studies are required to ascertain the disease phase and baseline characteristics of individuals who may benefit most from colchicine administration.

Canakinumab

Other medications directly targeting more specific cytokines in this inflammatory pathway such as IL-1β have been proposed, and canakinumab is an anti-IL-1β monoclonal antibody. The CANTOS trial was a randomised double-blind study, which randomised 10,061 participants with a previous myocardial infarction and an elevated hs-CRP (≥2 mg/L) to receive one of three doses of canakinumab, or a placebo, with a dose of 150 mg every three months. The trial demonstrated a significant reduction in the composite primary endpoint of nonfatal MI, nonfatal stroke, or cardiovascular death, though there was no significant difference in all-cause mortality, but a higher incidence of fatal infection in the canakinumab group [86].

Tocilizumab

Tocilizumab is a humanised anti-human IL-6 receptor antibody that results in IL-6 signalling inhibition [87]. Two small trials have shown that tocilizumab, when given in the context of ACS pre-PCI, attenuates the hs-CRP response and troponin release, and also increases the myocardial salvage index measured by cardiac MRI [88,89]. Interestingly, it appears Tocilizumab achieves this effect without inhibition of endothelial cell activation measured by an unchanged coronary flow reserve, despite an increase in a marker of endothelial cell activation, vascular cell adhesion molecule (VCAM)-1, suggesting an independent mechanism that has not yet been elucidated [90]. Further investigation with proteomic analysis has demonstrated potential mechanisms, such as an increased level of proteinase 3 following tocilizumab administration, suggesting neutrophil apoptosis which leads to neutropaenia; the suppression of C-C motif chemokine ligand (CCL)-23, which plays a role in leukocyte trafficking,; and a reduction in numerous other acute phase proteins, such as hepcidin, insulin-like growth factor-binding protein (IFGBP)-4, and lipopolysaccharide-binding protein [91].

Ziltivekimab

Colchicine should be used with caution in individuals with chronic kidney disease (CKD). The RESCUE trial was a randomised double-blind phase 2 trial including individuals with moderate-to-severe CKD and a hs-CRP of at least 2 mg/L, and randomised 264 participants to received either Ziltivekimab 7.5 mg, 15 mg, or 30 mg every 4 weeks up to 24 weeks vs. placebo, and it found that there was a significant reduction in hs-CRP as well as other biomarkers of inflammation and thrombosis [92]. ZEUS trial is an ongoing randomised trial randomising patients with CKD and established CAD, with an elevated baseline hs-CRP, to receive either human monoclonal antibody targeting IL-6 ligand Ziltivekimab or a placebo, with a composite primary outcome of time to first MACE, and the results are eagerly anticipated [93].

IL-1 targeting therapies

A Phase II trial of 182 patients randomised to receive 14 days of IL-1 receptor antagonist anakinra, or a placebo, following non-ST-elevation ACS, showed that while there was a significant reduction in IL-6 and hs-CRP levels after 14 days, there was an excess of MACE in the treatment arm at 1 year [94].

Goflikicept traps both Il-1a and IL-1B as well as binding to the IL-1 receptor antagonist, and a small trial of 102 patients presenting with STEMI, randomised to receive either goflikicept 80 mg, goflikicept 160 mg, or placebo, found that there was a significant reduction in hs-CRP after 28 days [95].

Methotrexate

Not all therapies targeting inflammation have shown promising results from the perspective of cardiovascular outcomes. The CIRT trial was a randomised double-blind trial, which randomised 4786 patients with previous MI or multivessel CAD, who also had either type 2 diabetes or metabolic syndrome, to receive either low-dose methotrexate or placebo, and after a median follow up of 2.3 years showed no reduction in levels of IL-1β, IL-6, and CRP, or any significant reduction in a primary endpoint of non-fatal MI, nonfatal stroke, cardiovascular death, or hospitalisation for unstable angina requiring revascularisation [96]. The anti-inflammatory action of methotrexate likely occurs via multiple mechanisms, not only the inhibition of dihydrofolate reductase but also the inhibition of purine and pyrimidine synthesis, the promotion of adenosine release, and multiple other potential pathways [97].

## 6. Future Directions

This narrative review has outlined the recent advances in understanding the importance of anti-inflammatory pathways in the development of atherosclerosis, and how this has impacted a number of potential treatment options. Within the limitations of a narrative review as opposed to a systematic review or meta-analysis, it is nevertheless clear that, up to this point, only colchicine has demonstrated positive results of significance to change guideline recommendations, though multiple pre-clinical studies have identified future potential therapeutic targets.

Well-established secondary preventative therapies are still relevant and should not be forgotten, such as lipid-lowering therapies with the monitoring of LDL-C targets and combination therapies including statins, ezetimibe and bempedoic acid or PCSK9i, but with growing recognition of the importance of inflammation and additional drug targets; subsequent therapeutic options based on individual risk assessment should guide treatment [98]. This risk assessment may centre around the identification of therapeutically relevant inflammatory biomarkers; for instance, hs-CRP is a well-established marker of residual inflammatory risk [11] but it is not clear whether routine measurement should guide anti-inflammatory therapy. Similarly, the potential for risk profiling based on other biomarkers, such as IL-6, IL-1β, or other markers in early research, such as intermediate monocytes, while biologically plausible and attractive conceptually, requires further research to establish potential clinical relevance in risk stratification.

One focus of therapeutics may be in targeting chemokines, for example CC-chemokine ligand 2 (CCL-2)/CC-chemokine receptor 2 (CCR-2), which are involved in recruiting myeloid cells to atherosclerotic lesions [99]. There is a significant association with CCL2 levels and atherosclerosis, and a meta-analysis of 14 studies utilising a mouse model found that pharmacological therapies targeting CCL-2 or CCR-2 reduced the size of atherosclerotic lesions; however, there is a highlighted need for future high-quality studies [100]. The NLRP3 inflammasome is another potential target, with various inhibitors having been identified to regulate NLRP3 transcription, translation, and activation [101]. One such recent example is a novel bispecific antibody targeting the IL-1R1 and also inhibiting the NLRP3 inflammasome, which not only slows the development of atherosclerotic plaques in a mouse model, but also improves plaque stability [102]. The adaptive immune response also provides a potential therapeutic target, with an understanding of the importance of T cells (Tregs) in the development of atherosclerosis [103]. Targeting Tregs provides an attractive treatment option, as they are not only involved in the IL-1 and IL-6 inflammatory pathways, but are also involved in multiple other mechanisms which may confer a greater benefit due to the many inflammatory pathways implicated in the development of CAD [104]. Anti-oxidised LDL Chimeric antigen receptor (CAR) Tregs have been developed and have shown reduced formation of macrophage foam cells in vitro and a reduction in atherosclerotic plaque formation in a mouse model [105]. Sodium–glucose co-transport 2 inhibitors (SGLT2is) have already demonstrated clinical benefit in novel areas of cardiovascular disease such as heart failure outcomes in cancer patients; several pre-clinical and some early clinical studies have also demonstrated a potential anti-inflammatory and atheroprotective effect, which requires further investigation in larger-scale randomised controlled studies [106,107].

Periprocedural inflammation during PCI is another important target. Colchicine has had conflicting results thus far with regard to periprocedural myocardial injury, though it does reduce NETs, expression of inflammatory cytokines such as IL-6, and CRP [63,83,84]. There is a need to further investigate its effect on other markers of inflammation, such as other cytokines or chemokines in the periprocedural setting, as well as its effect on differential expression of monocyte subsets.

Future studies investigating these novel therapeutic targets may therefore identify patients with higher individualised risk using established biomarkers such as hs-CRP as well as emerging novel biomarkers such as intermediate monocytes, Tregs, IL-6, and pentraxin-3 as those who may gain the most benefit from routine anti-inflammatory therapy periprocedurally in the context of PCI. Trials should focus on clinical endpoints such as type 4a myocardial infarction as well as overall MACE, as well as other markers of inflammation such as cardiac magnetic resonance imaging measures of infarct size and serial biomarker measurements. Further work is also needed to identify whether routine anti-inflammatory therapy in procedures that have been shown to confer a higher inflammatory risk, such as coronary bifurcation or in-stent restenosis, may provide a clinical benefit. Finally, a longer-term follow up is also required to assess the modulation of residual inflammatory risk following anti-inflammatory therapy and PCI, both in terms of clinical outcomes and biomarker expression.

## 7. Conclusions

In conclusion, there are a number of ‘take home messages’ for practitioners performing PCI. Firstly, while the identification of the importance of the residual risk of inflammation in the development of CAD and acute inflammation in the context of ACS has led to guideline-altering therapy in colchicine, other novel therapeutic agents have not yet been identified, and there is a significant treatment gap with regard to targeted anti-inflammatory therapies. Secondly, although the importance of inflammation in the periprocedural PCI setting is well established, the question of routine anti-inflammatory therapy in this context remains unanswered. Therefore, there is a need for future studies to determine the patient group that may gain the most clinical benefit from routine treatment with anti-inflammatory medication long-term after PCI, which may require biomarker identification for risk stratification as well as specific procedural characteristics, but also the specific drug target and anti-inflammatory therapy, whether that be colchicine or a novel therapeutic agent not yet currently established in guideline recommendations. Finally, in the growing age of precision medicine, answering these questions will be vitally important in improving both acute and long-term adverse outcomes related to peri-procedural inflammation in PCI.

## Figures and Tables

**Table 1:** Summary of relevant studies investigating the use of colchicine across chronic and acute coronary syndromes, and PCI. MI: myocardial infarction; CV: cardiovascular; PCI: percutaneous coronary intervention; STEMI: ST-elevation myocardial infarction; IHD: ischaemic heart disease; ACS: acute coronary syndrome; MACE: major adverse cardiovascular events; HR: hazard ratio.

Study	Population (*n*)	Intervention and Timing	Median Follow Up	Primary Endpoint	Effect on Inflammatory Biomarkers	Effect on Clinical Outcome
COLCOT	Recent MI within 30 days (*n* = 4745)	Colchicine 0.5 mg daily vs. placebo; initiated within 30 days of MI	22.6 months	Composite of death from CV causes, resuscitated cardiac arrest, MI, stroke, or urgent hospitalisation for angina resulting in coronary revascularisation	No significant difference in hs-CRP or leukocyte counts	Reduction in primary endpoint: 5.5% vs. 7.1% (HR 0.77, 95% CI 0.61–0.96)
COLCOT secondary analysis	COLCOT population (*n* = 4661)	Colchicine 0.5 mg once daily vs. placebo, initiated ≤3 days, 4–7 days, or ≥8 days after MI	22.7 months	Composite of CV death, resuscitated cardiac arrest, MI, stroke, or urgent hospitalisation for angina requiring coronary revascularisation	Not reported	Reduction in primary endpoint only in patients in whom colchicine was initiated ≤ day 3 compared with placebo: 4.3% vs. 8.3% (HR 0.52, 95% CI 0.32–0.84)
CLEAR	Acute MI treated with PCI (*n* = 7062)	Colchicine 0.5 mg once daily vs. placebo; within 72 h of PCI	3 years	MACE, composite of CV death, MI, stroke, or ischaemia-driven revascularisation	Reduced hs-CRP at 3 months	No significant reduction in primary endpoint: 9.1% vs. 9.3% (HR 0.99, 95% CI 0.85–1.16, *p* = 0.93)
LoDoCo2	Chronic coronary disease clinically stable for ≥6 months (*n* = 5522)	Colchicine 0.5 mg once daily vs. placebo; long-term therapy	28.6 months	Composite of CV death, MI, ischaemic stroke, or ischaemia-driven revascularisation	Not reported	Reduction in primary endpoint 6.8% vs. 9.6% (HR 0.69, 95% CI 0.57–0.83, *p* < 0.001)
COPE-PCI	Stable angina or non-STEMI undergoing PCI (*n* = 75)	Colchicine 1.5 mg total dose vs. placebo; 1 mg followed by 0.5 mg after 1 h, 6–24 h pre-PCI	24 h post-procedure	Periprocedural MI	No difference in hs-CRP, reduction in post-PCI leukocyte count in NSTEMI	No periprocedural MI in either group. Lower hs-troponin-I rise (59 vs. 166, *p* = 0.02), and periprocedural myocardial injury
Colchicine-PCI	Suspected IHD/ACS referred for angiography ± PCI (*n* = 400)	Colchicine 1.8 mg total dose vs. placebo; 1.2 mg followed by 0.6 mg 1 h later or immediately pre-procedure	30 days	PCI-related myocardial injury	Attenuated rise in IL-6 and hs-CRP 24 h post-PCI	No difference in PCI-related myocardial injury (57.3% vs. 64.2%) or 30-day MACE (11.7% vs. 12.9%)
PodCAST-PCI	STEMI undergoing PCI (*n* = 321)	Colchicine 1.5 mg total dose vs. placebo; 1 mg immediately pre-PCI followed by 0.5 mg 24 h later, then once daily until discharge from hospital	1 year	Coronary no-reflow following PCI	No significant reduction in hs-CRP, troponin, or P-selectin	No significant difference in primary outcome (*p* = 0.98)

## Data Availability

No new data were created or analysed in this study. Data sharing is not applicable to this article.

## Abbreviations

The following abbreviations are used in this manuscript:

CVD	Cardiovascular disease
PCI	Percutaneous coronary intervention
IL	Interleukin
hs-CRP	High-sensitivity C-reactive protein
CAD	Coronary artery disease
LDL-C	Low-density lipoprotein cholesterol
MACE	Major adverse cardiovascular events
NLRP3	Nucleotide binding oligomerization domain-like receptors pyrin domain-containing 3
ACS	Acute coronary syndromes
BMS	Bare metal stent
MI	Myocardial infarction
NSTEMI	Non-ST elevation myocardial infarction
STEMI	ST elevation myocardial infarction
DCB	Drug-coated balloon
NET	Neutrophil extracellular trap
LVEF	Left ventricular ejection fraction
PCSK9	Proprotein convertase subtilisin/kexin type 9
ACEi	Angiotensin-converting enzyme inhibitor
ARB	Angiotensin-1 receptor blocker
VCAM	Vascular cell adhesion molecule
CCL	CC motif chemokine ligand
CCR	CC motif chemokine receptor
IGFBP-4	Insulin-like growth factor-binding protein 4
CKD	Chronic kidney disease
SGLT2i	Sodium–glucose cotransporter-2 inhibitors
CAR	Chimeric antigen receptor
